# Endophilin recruitment drives membrane curvature generation through coincidence detection of GPCR loop interactions and negative lipid charge

**DOI:** 10.1074/jbc.RA120.016118

**Published:** 2020-12-06

**Authors:** Samsuzzoha Mondal, Karthik B. Narayan, Imania Powers, Samuel Botterbusch, Tobias Baumgart

**Affiliations:** Department of Chemistry, University of Pennsylvania, Philadelphia, Pennsylvania, USA

**Keywords:** GPCR, adrenergic receptor, endophilin, BAR domain, endocytosis, membrane curvature, BAR, BIN/Amphiphysin/Rvs, CD, circular dichroism, CIE, clathrin-independent endocytosis, CME, clathrin-mediated endocytosis, DOPC, 1,2-dioleoyl-sn-glycero-3-phosphocholine, DOPS, 1,2-dioleoyl-sn-glycero-3-phospho-L-serine, FEME, fast endophilin-mediated endocytosis, FRAP, fluorescence recovery after photobleaching, GPCRs, G-protein-coupled receptors, GUVs, giant unilamellar vesicles, ITO, indium tin oxide, PC, phosphatidylcholine, PRDs, proline-rich domains, ROI, region of interest, SDP, sulfodichlorophenol, TEM, transmission electron microscopy, TIL, third intracellular loop, WPI, World Precision Instruments

## Abstract

Endophilin plays key roles during endocytosis of cellular receptors, including generating membrane curvature to drive internalization. Electrostatic interactions between endophilin’s BIN/Amphiphysin/Rvs domain and anionic membrane lipids have been considered the major driving force in curvature generation. However, the SH3 domain of endophilin also interacts with the proline-rich third intracellular loop (TIL) of various G-protein-coupled receptors (GPCRs), and it is unclear whether this interaction has a direct role in generating membrane curvature during endocytosis. To examine this, we designed model membranes with a membrane density of 1400 receptors per μm^2^ represented by a covalently conjugated TIL region from the β1-adrenergic receptor. We observed that TIL recruits endophilin to membranes composed of 95 mol% of zwitterionic lipids *via* the SH3 domain. More importantly, endophilin recruited *via* TIL tubulates vesicles and gets sorted onto highly curved membrane tubules. These observations indicate that the cellular membrane bending and curvature sensing activities of endophilin can be facilitated through detection of the TIL of activated GPCRs in addition to binding to anionic lipids. Furthermore, we show that TIL electrostatically interacts with membranes composed of anionic lipids. Therefore, anionic lipids can modulate TIL/SH3 domain binding. Overall, our findings imply that an interplay between TIL, charged membrane lipids, BAR domain, and SH3 domain could exist in the biological system and that these components may act in coordination to regulate the internalization of cellular receptors.

Endocytosis is an important regulatory pathway for G-protein-coupled receptor (GPCR)-mediated cell signaling processes (1, 2). Activated receptors can be downregulated through endocytosis and then either undergo lysosomal degradation or recycling back to the plasma membrane. This process modulates the number of available receptors on the plasma membrane (3, 4). During receptor endocytosis, multiple protein–protein and lipid–protein interactions at the membrane promote recruitment of endocytic accessory proteins and membrane invagination, followed by membrane scission (5, 6, 7). Understanding the orchestration of these interactions would help in identifying potential therapeutic targets to rectify misregulations of the receptors leading to pathological conditions (8).

The BIN/Amphiphysin/Rvs (BAR) superfamily protein endophilin is one of the key effectors in clathrin-mediated endocytosis (CME) and particularly in clathrin-independent endocytosis (CIE) (9, 10). The dimeric protein on the one hand facilitates membrane remodeling *via* the crescent-shaped BAR-domain dimer (referred to as N-BAR domain because of the presence of an N-terminal amphipathic helix). On the other hand, endophilin’s Src homology 3 (SH3) domain recruits other endocytic proteins such as synaptojanin (11) and dynamin (12) and interacts with several other proteins that contain proline-rich domains (PRDs) (13). The multifunctionality of endophilin has given rise to a major research interest in its role in CIE pathways such as fast endophilin-mediated endocytosis (FEME). Endophilin plays a central role in FEME by driving cargo recruitment, membrane curvature generation, and membrane scission (14, 15). It has been suggested that molecular interactions occur between the endophilin SH3 domain and the third intracellular loop (TIL) of several GPCR family members during their internalization through the FEME pathway (10, 16, 17). Still, it has remained unclear how membrane remodeling by endophilin is functionally coupled with protein–protein interactions mediated by the SH3 domain.

Endophilin’s ability to sense and generate membrane curvature has been studied extensively *in vitro* as well as *in vivo*. Full-length endophilin and even the N-BAR domain alone can tubulate membranes when recruited *via* electrostatic interactions in the presence of a large proportion of anionic phospholipids (18, 19, 20, 21, 22, 23). While binding to TIL of GPCRs provides an additional modality of membrane recruitment, its effect on membrane curvature generation has not been explored. In order to address this question, we specifically chose the TIL of the β1-adrenergic receptor (β1-AR) as it has been shown to bind the endophilin SH3 domain (14, 16). Interestingly, activation of β1-AR is known to trigger the FEME pathway in retinal pigmented epithelial cells, whereas β2-AR, another GPCR from the AR family whose TIL does not bind to endophilin, does not trigger FEME (14).

To study the effect of TIL–endophilin interaction on membranes *in vitro*, we created a model membrane system by covalently coupling a TIL peptide from β1-AR to the lipid bilayer. This approach bypasses reconstitution of the entire GPCR and allows to maintain a consistent surface density of the conjugated TIL peptide on the membrane. We chose a lipid composition consisting to 95% of zwitterionic phosphatidylcholine (PC) such that the electrostatic interactions of the membrane lipids with either TIL or the endophilin BAR domain were suppressed. We show that under such conditions, the membrane recruitment of endophilin requires membrane-coupled TIL. We first demonstrate that TIL-mediated interactions can recruit endophilin to the membrane *via* its SH3 domain and that these TIL/SH3 interactions can be inhibited through the presence of anionic lipids due to their competitive electrostatic interactions with the cationic TIL. Our results reveal that endophilin retains its curvature sensing and generation properties when recruited *via* its SH3 domain, which has implications for our understanding of how endophilin’s BAR domains interact with the membrane and are involved in receptor internalization processes.

## Results

### Covalent conjugation of TIL to the lipid bilayer

**Figure 1:**
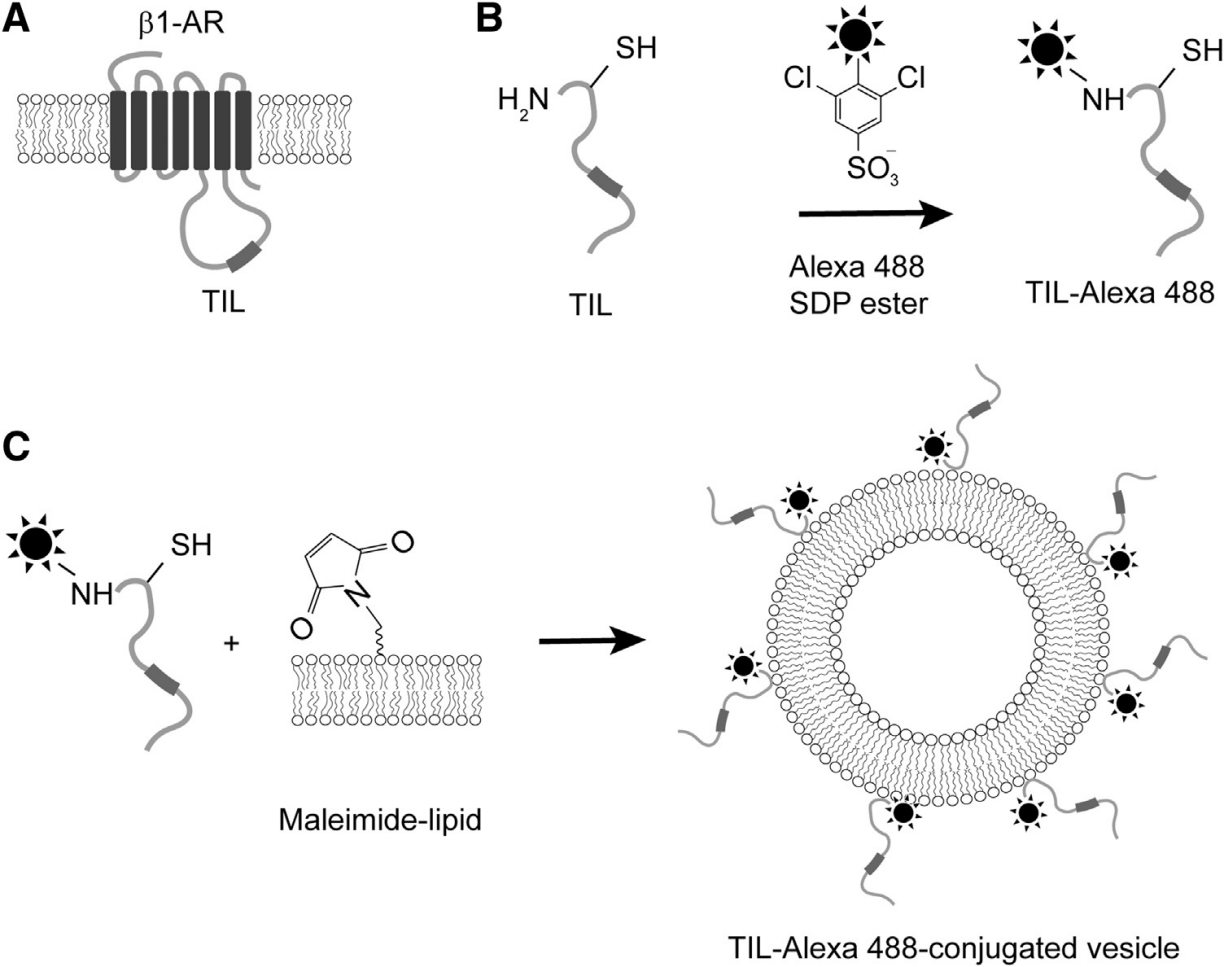
**Design of model membrane system by covalently linking a GPCR TIL to the lipid bilayer.***A*, cartoon representation of adrenergic receptor and position of TIL. *B*, labeling of TIL peptide with Alexa 488-SDP ester allows selective modification of the N terminus, leaving the cysteine side chain free. *C*, covalent conjugation of fluorescently labeled TIL to lipid bilayer containing lipids with maleimide-functionalized headgroup *via* cysteine–maleimide coupling reaction.

This project aimed to investigate the consequences of endophilin’s interaction with the β1-AR GPCR in a lipid model membrane system. Reconstitution of a full-length GPCR has been accomplished previously (24, 25, 26); however, complexity would be introduced since the GPCR-reconstitution process typically involves the use of detergents that interact with lipid bilayers and modify their properties (27). To circumvent these challenges, we covalently conjugated a known endophilin interaction domain, the receptor’s TIL (Fig. 1*A*), with lipid headgroups. The TIL contains an intrinsic cysteine residue (Cys261 in β1-AR) located near the N terminus of the TIL that allows formation of a covalent bond with a maleimide-derivatized phosphatidylethanolamine lipid headgroup. We hypothesized that such a covalent conjugation would attach TIL on the membrane in a way that would enable the PRD to interact with endophilin’s SH3 domain.

**Figure 2:**
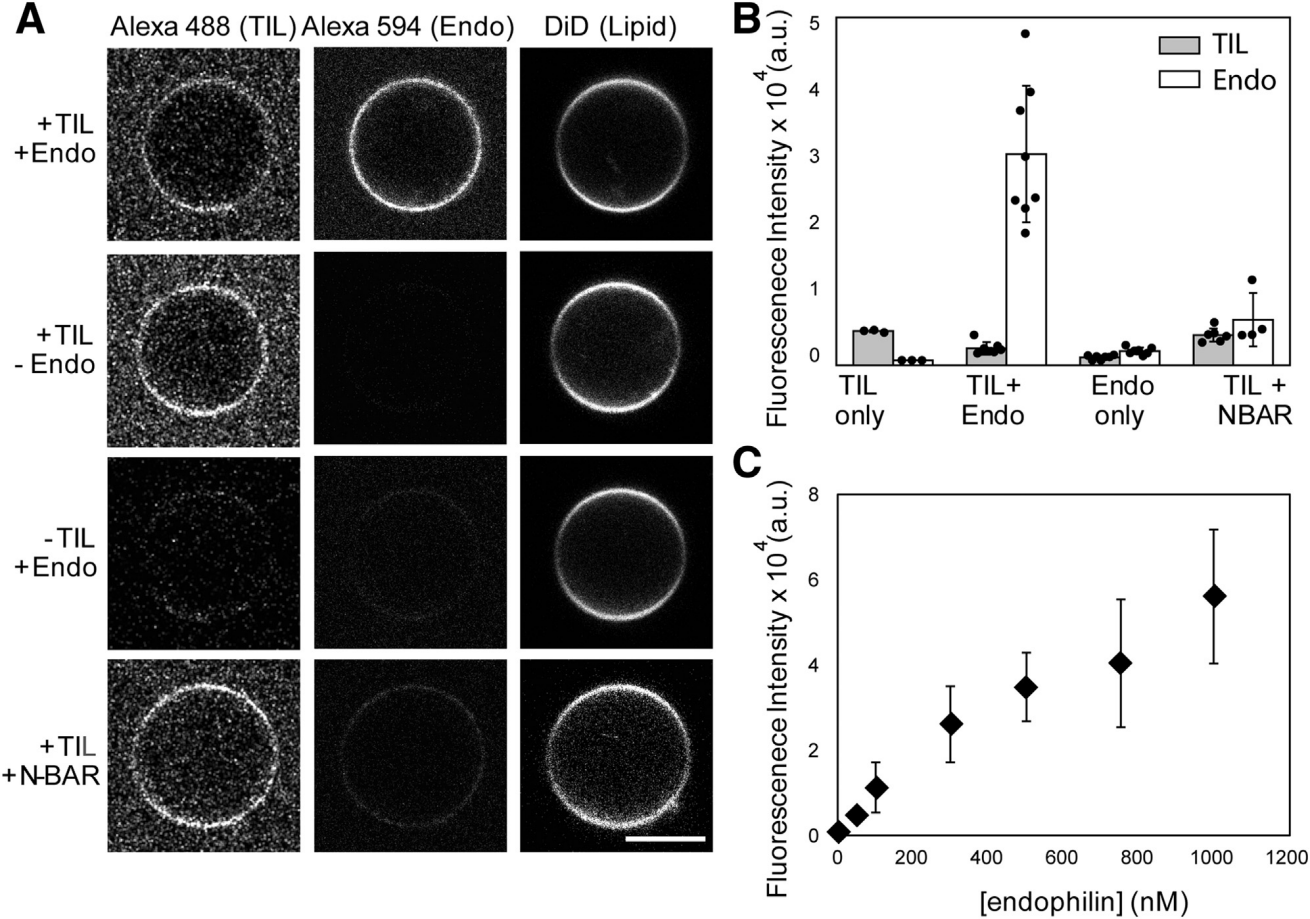
**Endophilin can be recruited to membranes in the absence of anionic lipids, through membrane-bound TIL.***A*, confocal images showing fluorescence channels corresponding to labeled TIL, endophilin (Endo) or its N-BAR, and of the membrane (through the DiD fluorophore). *First row*, demonstration of Alexa 594 labeled endophilin A1 binding to TIL-Alexa 488 conjugated vesicles (+TIL/+Endo). *Second row*, TIL-Alexa 488 conjugated GUVs in absence of endophilin (+TIL/−Endo). *Third row*: in the case of GUVs not conjugated to TIL (−TIL/+Endo), no significant recruitment of endophilin to the membrane is observed, as expected. *Last row*, unlike full-length endophilin, its N-BAR domain is not recruited to the TIL-conjugated membrane (+TIL/+NBAR). All GUVs shown are composed of 94.5 mol% of DOPC, 5 mol% of MCC-PE, and 0.5 mol% of DiD. Scale bar 5 μm. *B*, bar plot showing fluorescence intensities from the GUV images. Error bars represent standard deviation (s.d.), and the dots indicate the intensities from individual GUVs. *C*, a titration plot showing the fluorescence response from the TIL-conjugated GUVs in the Alexa 594 channel (Endo) as a function of endophilin concentration. Data points indicate mean ± s.d., N ≥ 3.

The purified TIL has a molecular weight of 8861 Da and is mostly disordered in nature (Figs. S1 and S2). Using an orthogonal chemical reaction strategy, we attached a fluorophore to the N-terminal amine group of TIL and the fluorescently labeled TIL to a maleimide lipid. A commercially available sulfodichlorophenol (SDP) ester derivative of the fluorophore reacts with the N-terminal amine group leaving the cysteine–SH group free (Fig. 1*B*). The reaction was performed at pH 7.0 because at this pH, amine reactive probes react with the N-terminal amine group selectively over lysine side chains (28). The molecular weight of the fluorophore conjugated TIL was confirmed by mass spectrometry (Fig. S3). Further, incubation of the peptide with giant unilamellar vesicles (GUVs) containing 5 mol% MCC-PE resulted in the coupling of the peptide on the membrane surface *via* cysteine–maleimide chemistry (Fig. 1*C*). We verified this attachment on the GUVs by observing Alexa 488 fluorescence from the GUV surface *via* confocal microscopy imaging (Fig. 2*A*). We estimated the surface density of TIL from the Alexa 488 fluorescence intensity following a method reported before (29, 30). We found the density to be 1400 ± 600 TIL molecules per μm^2^ membrane area, which is comparable with the range of densities (700–1200 per μm^2^) of various receptors such as IgE receptor, epidermal growth factor receptor, and lipoprotein receptors in cells (31, 32, 33). The average density of β-adrenergic receptors, however, is not more than 100 per μm^2^ (34).

We observed that the presence of 5 mol% MCC-PE resulted in an increase in the negative zeta potential (ζ) of the 1,2-dioleoyl-sn-glycero-3-phosphocholine (DOPC) bilayer from −3 mV (±1 mV) to −18 mV (±2 mV). A similar change in ζ was observed upon incorporation of 5 mol% 1,2-dioleoyl-sn-glycero-3-phospho-L-serine (DOPS) to DOPC membranes. However, after the conjugation of TIL, the zeta potential of 5 mol% MCC-PE vesicles was found to be −8 mV (±1 mV) because of the net positive charge of the TIL peptide (Table S1).

### TIL-SH3 interaction mediates recruitment of endophilin to lipid bilayers

Earlier studies have found that endophilin A1 binds to the TIL of β1-AR with a dissociation constant of 47 nM (16). This interaction has been hypothesized to be a key element in the trafficking of this GPCR upon activation. Consequentially, we asked if this strong binding interaction would lead to recruitment of endophilin to our TIL-conjugated membrane surface in the absence of anionic phospholipids (other than 5 mol% MCC-PE). A single cysteine mutant (E241 C, C108S, C294S, C295S) of rat endophilin A1 was labeled with Alexa 594-maleimide, in order to visualize the protein on GUVs through confocal microscopy imaging. When we mixed the TIL-conjugated GUVs with Alexa 594-labeled endophilin and imaged within 10 min of incubation, we did observe binding of endophilin to the GUVs. This observation implies that TIL recruited endophilin onto the membrane (Fig. 2, *A*–*B*). The extent of endophilin binding to the GUV surface with respect to the bulk endophilin concentration follows a binding isotherm pattern that reaches saturation beyond an endophilin concentration of 1 μM (Fig. 2*C*). However, it would be quite challenging to estimate the apparent dissociation constant from this data since in addition to being recruited to the membrane, endophilin interacts with the unbound TIL in the solution.

To confirm that the recruitment of the endophilin to the GUVs is solely *via* TIL but not due to the electrostatic interactions with negatively charged MCC-PE, we performed a control experiment with GUVs of the same lipid composition but not conjugated with TIL. We observed negligible binding of endophilin (Fig. 2, *A*–*B*), which implies that low negative charge contributions from 5 mol% of MCC-PE are not sufficient for direct recruitment of endophilin. It is noteworthy to mention here that earlier studies that reported direct recruitment of endophilin or its BAR domain to the membrane *via* electrostatic interactions used larger excess (40–75 mol%) of anionic phospholipids in the bilayer (18, 21, 23).

In order to further confirm that this binding is mediated by SH3–TIL interaction only, we tested an N-BAR only mutant of endophilin (that lacks the SH3 domain). The N-BAR mutant shows significantly less fluorescence intensity from the TIL-conjugated GUV surface again suggesting binding predominantly *via* the SH3 domain. The GUV imaging data confirm that endophilin can be recruited onto membranes by TIL under conditions that are not dominated by electrostatic interactions between the membrane and the BAR domain.

### TIL binds electrostatically to negatively charged membranes

**Figure 3:**
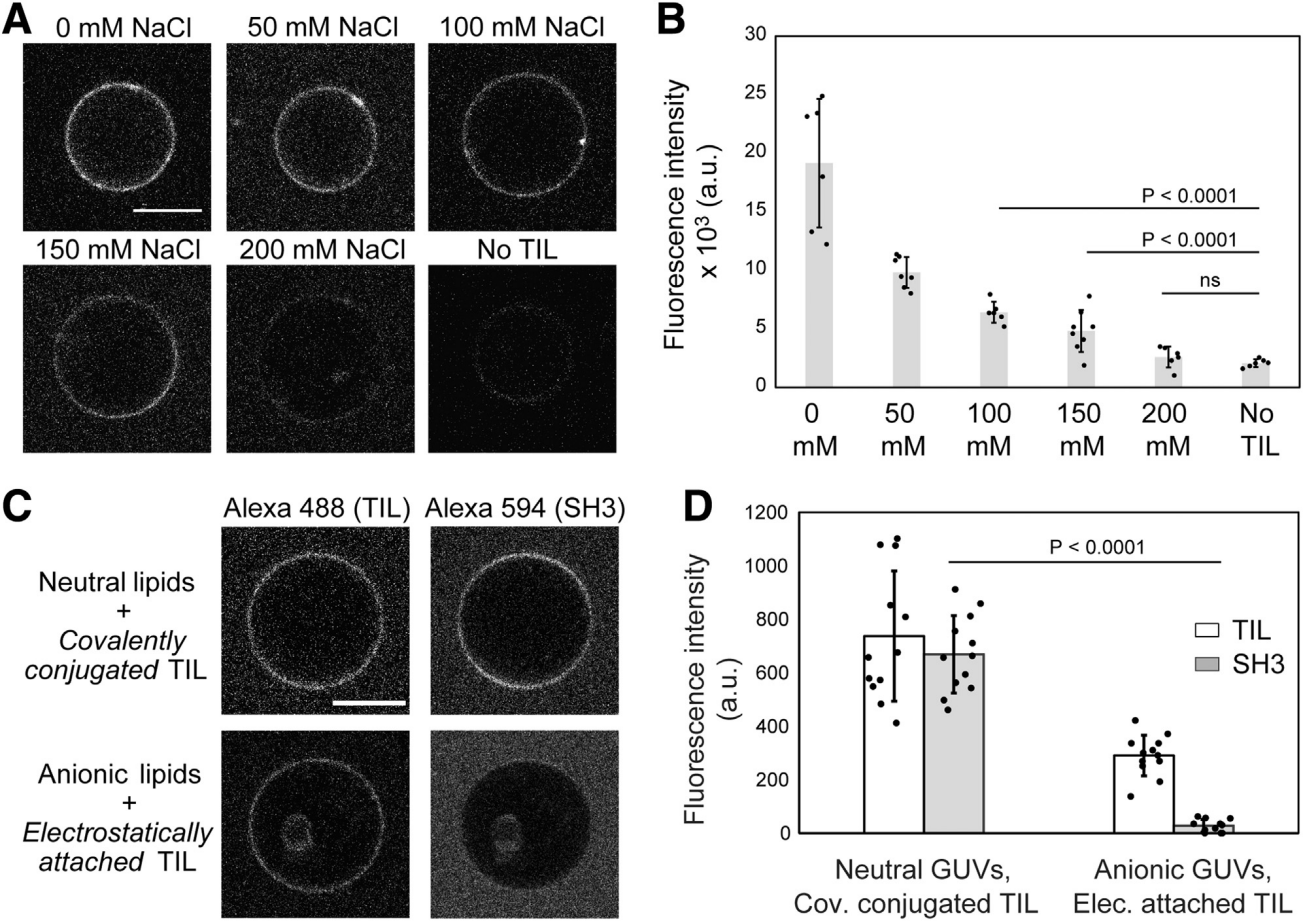
**TIL spontaneously binds to anionic lipid containing membranes, and the TIL–anionic lipid interaction interferes with SH3-domain recruitment.***A*, confocal images showing TIL-Alexa 488 fluorescence from the GUVs composed of DOPS/DOPE/DOPC (45:30:25). The ionic strength of the bulk solution was varied by changing the sodium chloride content in buffer (20 mM HEPES, pH 7.4) from 0 to 200 mM. *B*, bar plot showing the fluorescence intensity of bound TIL-Alexa 488 on GUVs at various salt concentrations. A decrease in the binding with increasing ionic strength suggests that the nature of binding is electrostatic. *C*, confocal images showing recruitment of TIL and SH3 on GUVs at physiological salt concentration (150 mM) when TIL is covalently conjugated to GUVs composed of neutral lipids (MCC-PE/PC 5:95) *versus* when TIL is electrostatically attached to GUVs containing anionic lipids (PS/PE/PC 45:30:25). *D*, bar plot showing fluorescence intensities in the Alexa 488 (TIL) channel and Alexa 594 (SH3) channel from the GUV surfaces, which indicates that at 150 mM salt concentration covalently coupled TIL recruits the SH3 domain, whereas electrostatically attached TIL does not. All scale bars are 10 μm. Bar plots are represented as mean ± s.d. where individual GUV intensities are shown as dots. *p* values were obtained from Student’s *t*-test, N ≥ 6 for 3B and N ≥ 11 for 3D.

The TIL of β1-AR contains several lysine and arginine residues, which result in a highly positive net charge, with pI ∼12. Such highly cationic segments are likely to interact with the anionic lipid headgroups within the inner leaflet of the plasma membrane. In order to test this hypothesis, we studied the binding of TIL with GUVs consisting of a lipid mixture designed to mimic the negatively charged inner leaflet of the plasma membrane (PS/PE/PC 45:30:25). When we imaged the GUVs in the presence of Alexa 488-labeled TIL through confocal fluorescence microscopy, TIL showed binding to the GUVs (Fig. 3*A*). In order to ask if this mode of binding is primarily electrostatic, TIL and GUVs were mixed under various ionic strength conditions generated by changing the NaCl content of the mixing buffer in the range of 0 to 200 mM (Fig. 3, *A*–*B*). A steady increase in the extent of binding with a decrease in ionic strength results from the nonlinear dependence of the electrostatic binding free energy on the salt concentration (35, 36, 37). This data confirmed that the major mode of the TIL–lipid bilayer interaction is primarily electrostatic in nature.

From the ionic-strength-dependent binding studies, we learned that TIL shows a weak but significant membrane interaction in the range of physiological salt concentration (150 mM NaCl). GPCRs are known to bind anionic headgroup containing phospholipids, and this binding interaction is believed to have roles in GPCR activation and G-protein binding (38, 39, 40). Competitive membrane binding interactions could significantly influence the binding of TIL to its protein binding partners *in vivo*. Therefore, we asked if the TIL–SH3 interaction would be influenced by the interactions of TIL and anionic lipid headgroups. For this experiment, we used the endophilin-SH3 domain since the full-length endophilin would bind to the negatively charged membranes irrespective of TIL–SH3 interactions. The purified SH3 domain was labeled at the N-terminus with Alexa 594-NHS ester for fluorescence microscopy imaging. Interestingly, the SH3 domain did not show detectable binding to the GUV surface with electrostatically bound TIL in the presence of 150 mM NaCl. In contrast, TIL conjugated to neutral membranes allowed recruitment of SH3 under the same salt concentration (Fig. 3, *C*–*D*). Our data therefore indicate that the TIL–membrane interaction could be a crucial regulator of SH3-domain-mediated endophilin recruitment *via* TIL.

### Endophilin tubulates neutral membranes when recruited by TIL

Endophilin has been widely investigated for its capacity to tubulate lipid bilayers *in vitro* and when overexpressed in live cells. Both full endophilin and the N-BAR domains are known to tubulate LUVs composed of anionic phospholipids, as demonstrated earlier with transmission electron microscopy (TEM) (18, 19). Mechanisms of curvature generation by BAR-domain proteins that have been suggested include 1) scaffolding—generation of a 3D protein assembly upon oligomerization of the membrane-bound BAR-proteins that modulate membrane shape (41); 2) amphipathic helix insertion—embedding of the N-terminal amphipathic helix present in the N-BAR subfamily proteins resulting in membrane bending (20); and 3) protein crowding—enhancement of membrane spontaneous curvature in order to reduce the steric pressure generated by crowded membrane anchored proteins (42, 43). These mechanisms are based on observations that showed that these proteins primarily anchor to the membrane *via* the N-BAR domain that forms strong electrostatic interactions with the anionic lipid headgroups. We therefore asked whether membrane curvature could be induced by recruitment of endophilin to the membrane primarily through the SH3-domain-mediated interaction.

**Figure 4:**
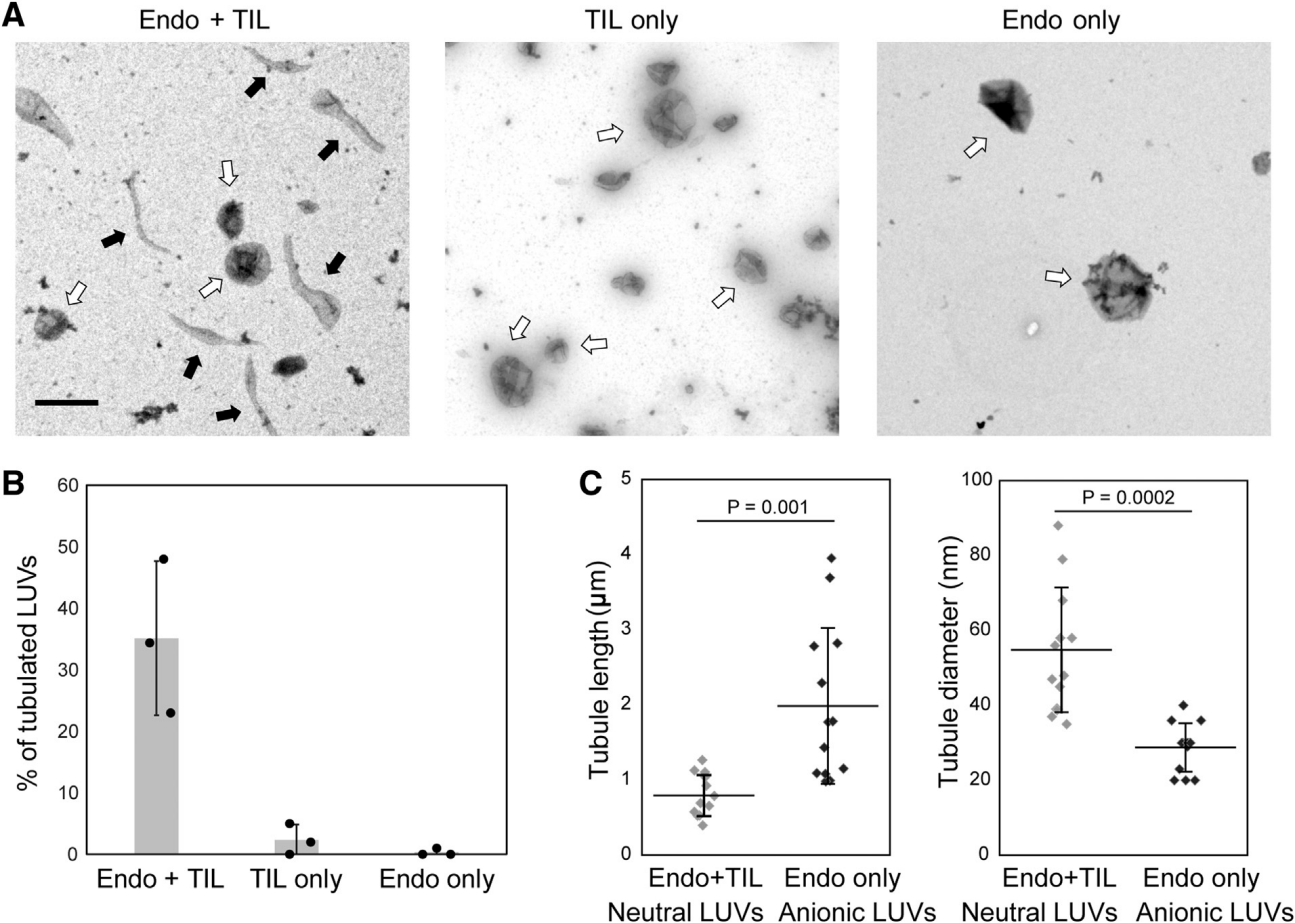
**Endophilin tubulates TIL-conjugated LUVs even in the absence of anionic phospholipids.***A*, TEM images of LUVs composed of 5% MCC-PE and 95% DOPC conjugated with TIL show tubulation in the presence of endophilin (left panel, extended data set in Fig. S6) whereas there is no tubulation in case of TIL-conjugated LUVs in the absence of endophilin (middle panel). Conversely, endophilin itself does not tubulate LUVs that are not conjugated with TIL (right panel). *White arrows* indicate intact LUVs, whereas *black arrows* indicate tubulated LUVs. Scale bar 500 nm. *B*, extent of tubulation shown as percentage of tubulated vesicles over all the vesicles imaged. Bar graph indicates mean ± s.d. percentage of tubules observed in three independent trials. Each *black circle* represents the average percentage of tubules obtained from all the images collected during an individual trial. *C*, scatter plots showing the distribution of tubule length and diameter formed by endophilin with TIL-conjugated LUVs (*gray*) and with LUVs composed of anionic phospholipids (DOPS:DOPE:DOPC 45:30:25) (*black*). The horizontal lines indicate mean values of the distributions, and the whiskers indicate standard deviations. *p* values were obtained from Student’s *t*-test, N = 13.

Our confocal studies with TIL-conjugated GUVs however did not show any resolvable tubulation in the presence of endophilin. One possibility for this might be that the formed tubules were too short to be visualized by an optical microscope. Therefore, we performed a tubulation study with TEM. We prepared LUVs of 400 nm diameter (Fig. S4) containing maleimide lipids, to which we covalently coupled TIL, followed by incubation with endophilin. Interestingly, TEM images show that 35% of the TIL-conjugated LUVs form tubules in the presence of endophilin (Fig. 4 and Fig. S6). Only about 2% of the TIL-functionalized LUVs show tubulation in the absence of endophilin. LUVs having the same lipid composition without TIL conjugation do not show any tubulation in the presence of endophilin (Fig. 4, *A*–*B*), indicating that the presence of MCC-PE alone does not contribute to the tubulation.

We determined the tubule diameters from the TEM images in the case of TIL-recruited endophilin and compared them to tubules obtained in the case of endophilin recruited by electrostatic interactions to membranes containing 45 mol% anionic phospholipids (Fig. 4*C*, and Fig. S5). Identical endophilin:lipid ratios and incubation times were maintained for both cases. Tubule diameters were in the range of 20 to 40 nm, comparable with earlier reports, in the case of highly anionic LUVs where endophilin was recruited by electrostatic interactions (20). On the other hand, tubule diameters were significantly larger, in the range of 40 to 80 nm, in the case of TIL-recruited endophilin (Fig. 4*C*). Furthermore, the majority of tubules generated by TIL-recruited endophilin were below 1 μm in length, whereas the tubules formed in the case of anionic-lipid-recruited endophilin were significantly longer and in the range of 1 to 5 μm (Fig. 4*C*, and Fig. S5). The tubule length below 1 μm supports our hypothesis that the tubules formed are indeed too short to be observable through confocal microscopy. The observations from TEM imply that the curvature generation is higher for the electrostatically recruited endophilin onto membranes having a large proportion of negatively charged lipids. However, endophilin recruited to membranes through TIL also induces membrane tubulation.

### TIL-recruited endophilin is curvature-sorted on membranes

**Figure 5:**
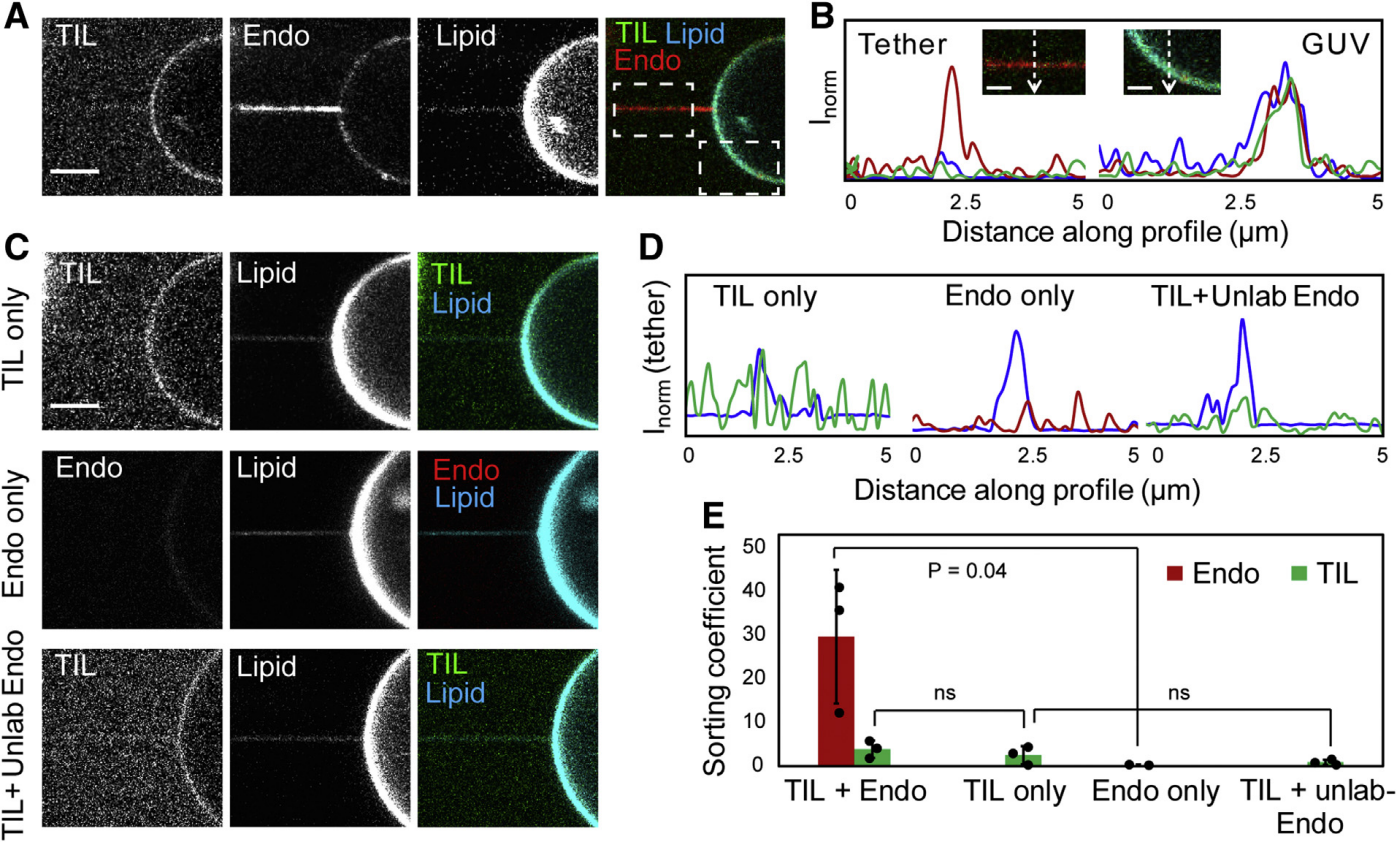
**Curvature sorting of TIL-recruited endophilin observed in membranes with neutral net charge.***A*, confocal fluorescence images showing a membrane tether pulled from a GUV conjugated to TIL-Alexa 488 in the presence of endophilin-Alexa 594. Images are recorded in three different channels to show the distribution of TIL, endophilin, and the lipid dye DiD on the membrane tether and on the flatter GUV area. In the endophilin channel, the tether shows higher fluorescence intensity compared with the vesicle, indicating curvature sorting of the protein. Scale bar 5 μm. *B*, intensity profiles of endophilin (*red*), TIL (*green*), and DiD (*blue*) on the membrane tether and on the GUV surface along the white lines shown in the insets (see *dashed white boxes* for their location in *A*, bars 2.5 μm). Higher intensity in the *red channel* from the tether indicates endophilin is enriched in the membrane tether region compared with the flatter GUV area. *C*, confocal images showing tether pulled from a TIL-Alexa 488 conjugated GUV in the absence of endophilin (TIL only), top panel; from a GUV having no TIL-Alexa 488 conjugated in the presence of endophilin (Endo only), middle panel; and from a TIL-Alexa 488 conjugated GUV in the presence of unlabeled endophilin (TIL+unlab Endo), bottom panel. Scale bar 5 μm. *D*, Intensity profiles across the tether region of the images shown in *C*. *E*, sorting coefficients for endophilin and TIL as quantified from the corresponding fluorescence images under the conditions mentioned in *B*–*C* using the formula described in the experimental section. Bar plots show the mean values ±s.d. of sorting coefficients obtained in three independent trials. Each black circle represents the average value of sorting coefficients obtained from all membrane tethers pulled during an independent trial. *p* values were obtained from Student’s *t*-test, N = 3.

BAR domain proteins sense membrane curvature through preferential partitioning into highly bent membrane regions (22, 44, 45, 46). Endophilin has been shown to be recruited at the sites of membrane invagination during CIE and to promote membrane scission (15). Our previous studies have shown that endophilin N-BAR is sorted onto membrane tethers pulled from GUVs containing a large amount (45 mol%) of anionic phospholipids (19, 22). Next, we asked if this curvature sorting behavior of endophilin is observed when the protein is recruited *via* its SH3 domain but not the BAR-domain. Incorporation of a small (0.2 mol%) amount of lipids containing a biotin-functionalized headgroup facilitated the pulling of membrane tethers from GUVs with the help of streptavidin-functionalized beads (22). The tethers formed were of approximately 70 ±30 nm in diameter and thus were within the range of the membrane invaginations formed during CIE that are 50 to 100 nm in diameter (10, 47). When tethers were pulled from TIL-conjugated GUVs in the presence of endophilin, we observed significantly higher fluorescence intensities of endophilin on tethers compared with the flatter membrane surface on the GUVs, indicating curvature sorting of endophilin (Fig. 5*A*, *B* and *E*).

Tethers pulled from TIL-conjugated GUVs in the absence of endophilin also did not show any curvature sorting of TIL (Fig. 5*C* top panel, Fig. 5, *D*–*E*). We calculated the membrane area coverage by TIL at the obtained average membrane density (∼1400 μm^−2^) by estimating the hydrodynamic radius for intrinsically disordered proteins (48). The membrane area coverage was found to be ∼3.5%, which is well below the threshold protein coverage for membrane bending by the crowding effect (∼20%) (49). This implies that in our experiments the TIL density on the membrane is not high enough to cause curvature sorting of TIL due to the crowding effect that has been reported for large, intrinsically disordered protein domains (50, 51). These data also show that the accumulation of endophilin on tethers is not due to curvature sorting of TIL.

Notably, no curvature sorting of TIL was observed in the presence of endophilin either (Fig. 5*A*, *B*, and *E*). One possibility behind not observing curvature sorting in the TIL channel could be loss of Alexa 488 fluorescence signal due to energy transfer to the Alexa 594-labels on neighboring endophilin molecules. To exclude this possibility, we pulled tethers from TIL-conjugated GUVs in the presence of unlabeled endophilin (Fig. 5*C* bottom panel). No curvature sorting of TIL was observed in this case either (Fig. 5, *D*–*E*). A second hypothesis is that the conjugated TIL is immobile on the membrane. We tested this second hypothesis through fluorescence recovery after photobleaching (FRAP) experiments that demonstrated recovery of TIL-Alexa 488 fluorescence from 60% to 90% in 20 s (Fig. S7). Therefore, TIL clearly is mobile on the membrane. Furthermore, photobleaching recovery studies on the membrane tether show recovery of the bleached Alexa 594 fluorescence indicating that the curvature-sorted endophilin is mobile on the tether (Fig. S8).

A third possibility could be that the intensity changes on the membrane tether caused by the curvature sorting of TIL is beyond our detection limit. One limitation of our system is the weak fluorescence signal from the membrane in the Alexa 488 channel due to low labeling efficiency (∼30%). This results in a weak diffraction-limited signal from the tether that is further masked by the background fluorescence from unbound TIL-Alexa 488 (Fig. 5, *A*–*D*). Therefore, it is likely that accurate determination of the TIL fluorescence intensities on tether, and therefore the sorting coefficient of TIL, was not possible in our experiments.

Finally, in the absence of TIL, no curvature sorting of endophilin was observed (Fig. 5*C* middle panel, Fig. 5, *D*–E). As we have shown that the mild negative charge contributions from the 5 mol% of MCC-PE are unable to recruit endophilin to the GUVs in the absence of TIL (Fig. 2, *A*–*B*), it also does not induce any detectable curvature sorting of endophilin.

Taken together, these findings strongly suggest that the curvature sensing capabilities of endophilin persist during its recruitment to the membrane by the SH3–TIL interaction even if the membrane is not rich in anionic phospholipids.

## Discussion

Electrostatic interactions between BAR domains and anionic lipid headgroups are considered essential not only for recruitment of BAR proteins to the membrane, but they also are critical for curvature sensing and generation of these proteins (20, 52, 53, 54, 55). In earlier studies on membrane curvature generation by endophilin and it’s N-BAR domain, 40 to 75 mol% of anionic phospholipids have been used in the lipid composition to facilitate protein enrichment to the membrane *via* the BAR domain (18, 21, 23, 53). Although the SH3 domain is known to mediate membrane recruitment of endophilin *via* protein–protein interactions, to what extent this recruitment pathway contributes to the curvature generation activity has so far remained unexplored. In our present contribution, we use membranes composed of 95% of zwitterionic lipids and only 5 mol% of the anionic lipid MCC-PE for functionalizing TIL, to ensure that endophilin recruitment to the membrane is driven primarily through SH3-mediated interactions. We show, at a biologically relevant TIL density on the membrane, that the TIL/SH3 interaction alone is sufficient to engage endophilin to the membrane to cause membrane tubulation as well as curvature sorting of the protein. It is likely that the anchoring of the BAR protein to the membrane *via* the SH3 domain enables various interactions to take place, such as engagement of the concave side of the crescent-shaped BAR domains to the membrane by short-range electrostatic and hydrophobic interactions with lipid molecules that together contribute to the curvature sensing and curvature generation effects we observe.

Membrane curvature generation can be easily facilitated by electrostatically recruiting BAR domains to the inner leaflet of the plasma membrane. In the FEME pathway, however, the activity of endogenous endophilin in cells is initiated by stimulation of GPCR followed by heterotrimeric G-protein release so that the GPCR’s TIL is available for endophilin binding (10, 14). From our study it is now clear that both BAR-domain and SH3-domain-mediated membrane anchoring can drive curvature sensing and membrane remodeling. Therefore, to cause membrane bending leading to receptor internalization, the cell must ensure that both interactions occur in place. Peripheral protein activities are known to be regulated by coincidence detection of membrane lipids as well as other protein binding partners on the membrane (56). Such proteins contain separate patches or domains to undergo protein–lipid and protein–protein interactions simultaneously. Similarly, one or more auxiliary protein-binding or lipid-binding domains are also found in various BAR proteins such as the SH3 domain in endophilin, amphiphysin, and many other BAR proteins, the PH domain in ASAP/ACAP family proteins, and PX domain in sorting nexins (57, 58). These additional domains enable BAR-domain containing proteins to sense changes in the protein–lipid microenvironment under stimulated conditions (58, 59). Therefore, we believe that, apart from membrane anchoring through N-BAR domains, coincidence detection of GPCR-TIL could play a key role in driving membrane curvature generation by endophilin.

The recruitment mechanism of endophilin to the plasma membrane is more complex than the binding mechanism in our current model membrane system where only TIL recruits endophilin to the membrane. In cells, an adaptor protein called lamellipodin contributes to endophilin clustering on the membrane, and through its multivalent binding it could establish a highly sensitive switch-like mechanism where, through cooperative binding, TIL/endophilin interactions are greatly amplified (10, 14). A more complete understanding of how endophilin present in lamellipodin-stabilized transient clusters interacts with TIL and drives membrane would require a more complex membrane system with reconstitution of both endophilin recruitment motifs of lamellipodin and TIL. This is currently work in progress in our lab.

Our finding that anionic lipid headgroups interfere with TIL binding to SH3 domains indicates that recognition of the activated GPCR by endophilin could be regulated by the lipid microenvironment, which acts a regulator of various protein–protein interactions including GPCR and its protein binding partners (40). We speculate that molecular interactions between BAR proteins, GPCR-TIL, and the phospholipid headgroups play an essential role in the downregulation of GPCRs by endocytic pathways.

## Experimental procedures

### Materials

The lipids DOPC, 1,2-dioleoyl-sn-glycero-3-phosphoethanolamine (DOPE), DOPS, 1,2-dipalmitoyl-sn-glycero-3-phosphoethanolamine-N-[4-(p-maleimidomethyl)cyclohexane-carboxamide] (16:0 MCC-PE), and 1,2-distearoyl-sn-glycero-3-phosphoethanolamine-N-[biotinyl(polyethylene glycol)-2000] (DSPE-PEG-Biotin) were obtained from Avanti Polar Lipids (Alabaster, AL). Alexa Fluor 488 SDP ester, Alexa Fluor 594 C5 maleimide, and the lipid fluorophore 1,1'-dioctadecyl-3,3,3',3'-tetramethylindodicarbocyanine, 4-chlorobenzenesulfonate salt (DiD), and BODIPY-FL-DHPE (N-(4,4-Difluoro-5,7-Dimethyl-4-Bora-3a,4a-Diaza-s-Indacene-3-Propionyl)-1,2-Dihexadecanoyl-sn-Glycero-3-Phosphoethanolamine, Triethylammonium Salt) were from ThermoFisher Scientific (USA). Streptavidin-conjugated microsphere beads (6 μm diameter) were from Polysciences (Warrington, PA). All common reagents used for the buffer preparation, such as HEPES, Tris, NaCl, Na_2_HPO_4_, NaH_2_PO_4_, ethylenediaminetetraacetic acid (EDTA), and dithiothreitol (DTT), were from Fisher Scientific (USA). β-casein from bovine milk was obtained from Sigma-Aldrich (USA). Unless otherwise specified, all chemicals from commercial sources were used without further purification.

### Construct design, protein expression, and purification of TIL

The TIL region of the β1-adrenergic receptor (amino acids 246–325) was cloned from a pcDNA3-Flag beta-1-adrenergic-receptor vector (Robert Lefkowitz lab, Addgene Plasmid #14698) by PCR using the following primers: forward 5′-AAAGGATCCCGGGTGTTCCGCG-3′ and reverse 5′-AAAGAATTCTTACGTCTTGAGCGCCTTCTG-3′). The TIL sequence was ligated into BamHI/EcoRI restriction sites of the pGEX6p1 vector for bacterial expression as an N-terminal GST-tagged protein.

The GST-tagged TIL was expressed in BL21-CodonPlus (DE3)-RIL competent cells (Agilent Technologies). Two 1 l cultures were grown from a 50 ml starter culture at 37 °C until the O.D. reached ∼0.8. The protein was induced with IPTG (300 μM), and the expression was carried out at 18 °C for 12 to 16 h. Cells were centrifuged at 5000 rpm for 10 min, and the pellets were resuspended in lysis buffer (50 mM tris, 300 mM NaCl, 1 mM EDTA, 2 mM DTT, pH 8.0), followed by addition of the protease inhibitor phenylmethylsulfonyl fluoride (1 mM). Cells were lysed by tip sonication, and centrifugation at 15,000 rpm for 60 min removed the cell debris. The supernatant was filtered through 0.22 μm pore size syringe filters (MilliporeSigma, Millex) and loaded onto a GST trap column (GE healthcare) with the lysis buffer. The bound GST-TIL was washed with the lysis buffer and eluted with a buffer containing 20 mM reduced glutathione along with 50 mM Tris, 300 mM NaCl, 1 mM EDTA, 2 mM DTT, pH 8.0. TIL was cleaved from the GST-tag by treating the eluate with PreScission protease (125 μg in 14 ml) at 4 °C for 4 to 6 h. The protease-treated protein mixture was loaded onto a HiTrap SP HP cation exchange column (GE Healthcare), and TIL was eluted by running a gradient of NaCl (buffer A: 20 mM sodium phosphates, 150 mM NaCl, 1 mM EDTA, 1 m TCEP, pH 7.0; buffer B: 20 mM sodium phosphates, 1 M NaCl, 1 mM EDTA, 1 mM TCEP, pH 7.0). Fractions were collected by monitoring the UV absorbance at 254 nm and analyzed *via* SDS-PAGE. Pure fractions containing TIL were combined and concentrated by Amicon ultra centrifugal filters with 3 kDa molecular weight cutoff (Millipore), and buffer was exchanged to either 20 mM HEPES, 150 mM NaCl, 1 mM TCEP; pH 7.4 (mentioned as HEPES-buffer hereafter) or phosphate buffer (10 mM sodium phosphate, 150 mM NaCl, 1 mM TCEP; pH 7.0) as required.

Molecular weight of the purified peptide was verified through MALDI mass spectrometry: expected m/z 8862 (M + H), observed m/z 8861.5 (Fig. S1). Purity of the peptide was confirmed with SDS-PAGE (Fig. S2*A*). The disordered nature of the peptide was verified by circular dichroism spectroscopy (Fig. S2*B*).

### N-terminal labeling of TIL

TIL was selectively labeled at the N-terminal amine group with Alexa 488-SDP ester. The peptide was buffer-exchanged to phosphate buffer (10 mM sodium phosphates, 150 mM NaCl, 1 mM TCEP; pH 7.0) in order to carry out the labeling reaction. The dye was added at a twofold molar excess to the peptide, and the reaction was carried out at 4 °C for 12 to 16 h. Progress of the reaction was monitored by MALDI mass spectrometer (m/z 9398 for TIL-Alexa 488), and the reaction was continued until 30 to 50% labeling of TIL was observed (Fig. S3). Excess fluorophore was separated from the peptide by passing the reaction mixture through a HiTrap desalting column (GE healthcare), eluted against HEPES buffer. Efficiency of the labeling was determined by measuring the fluorophore concentration (Alexa 488, ɛ_495_ 73,000 M^−1^ cm^−1^) *via* a nanodrop instrument (Thermo Fisher) and the peptide concentration *via* Bradford method.

### Characterization of TIL by MALDI-TOF-MS

MALDI mass spectrometry was performed to characterize purified TIL and to monitor progress of fluorophore labeling reactions (Figs. S1 and S3). About 1 μl of the peptide sample was mixed with 1 μl saturated solution of α-cyano-4-hydroxycinnamic acid (in 1:1 acetonitrile: water with 0.1% trifluoroacetic acid). The mixture was spotted onto a MTP 384 ground steel target plate (Bruker Daltonics) and allowed to dry. MALDI spectra were recorded with a Bruker UltrafleXtreme MALDI-TOF-MS (Bruker Daltonics).

### Circular dichroism (CD) spectroscopy

CD spectra were recorded with an AVIV CD spectrophotometer (Aviv Biomedical, NJ) using a High-Precision Quartz Suprasil cuvette (Hellma Analytics) at 25 °C. Typically, 5 μM TIL in 10 mM phosphate buffer containing 150 mM NaCl, pH 7.4 was used for recording CD spectra (Fig. S2*B*).

### Purification and labeling of full-length endophilin, N-BAR and SH3 domain mutants

Full-length rat endophilin A1 (with C108S, E241C, C294S, C295S mutations), its N-BAR domain (with C108S, E241C mutations) were expressed, purified, and labeled with Alexa 594-maleimide as described elsewhere (60). Briefly, proteins were expressed as GST fusions and purified using GST-affinity chromatography. GST tags were cleaved using PreScission protease, and the BAR proteins were further purified by anion exchange and size-exclusion chromatography techniques. Labeling at the cysteine residue positions was achieved by incubating the protein with Alexa 594-maleimide overnight at 4 °C.

The SH3 domain of rat endophilin A1 was also expressed as GST-fusion protein using a plasmid generously provided by Volker Haucke’s lab. The GST-tag was cleaved using thrombin protease, and the SH3 domain was further purified by anion exchange and size-exclusion chromatography techniques. The purified SH3 domain was labeled at the N terminus using Alexa 594-NHS ester following the same protocol used for N-terminal labeling of TIL as described in the previous section.

Protein concentrations were determined by measuring the absorbance at 280 nm (ɛ_280_ 17,545 M^−1^ cm^−1^) for unlabeled full-length endophilin. For all the other cases, the Coomassie (Bradford) Protein Assay (Thermo Scientific, USA) was used for determining protein concentrations.

### GUV preparation and conjugation of TIL to GUVs

GUVs were prepared by the electroformation method using indium tin oxide (ITO)-coated slides (61). Chloroform solutions of desired lipid composition were coated onto ITO slides and vacuum dried for at least 2 h. The lipid films were hydrated with sucrose solution (350 mOsm in MilliQ purified water). Electroformation was performed at 55 ºC for 1 h.

For TIL conjugation, GUVs composed of MCC-PE/DOPC/DiD (5:94.5:0.5 molar ratio) were mixed (in 1:10 GUV solution: buffer volumetric ratio) with TIL-Alexa 488 (100 nM) in HEPES buffer. The buffer osmolarity was preadjusted to the osmolarity of the sucrose solution used for electroformation. The mixture was incubated at room temperature for 6 to 8 h, and then the unreacted maleimide groups on the lipids were blocked by adding β-mercaptoethanol (5 mM).

### Confocal imaging and quantitative image analysis

Confocal images were recorded using a FluoView 3000 scanning system configured on an IX83 inverted microscope (Olympus, Center Valley, PA). Images were taken at room temperature using a 60 × 1.1 NA water immersion objective (Olympus). GUVs suspended in buffer were placed into the cavity of a home built imaging chamber formed by two glass coverslips (25 × 25 mm^2^, Fisher Scientific) held together with a glass holder with the help of vacuum grease. The bottom coverslip was passivated with β-casein solution (5 mg/ml) in order to avoid sticking of GUVs to the glass surface. GUVs settled on the bottom glass surface were imaged.

For study of the ionic-strength-dependent TIL binding to anionic lipid containing membranes, GUVs composed of DOPS/DOPE/DOPC/DiD (45:30:24.5:0.5 M ratio) were mixed with TIL-Alexa 488 in HEPES buffer containing 0 to 200 mM NaCl. The osmolarity difference between the inside and outside of the GUVs was balanced by adding requisite amounts of glucose to the outer solution. TIL-Alexa 488 binding to GUVs was determined by imaging *via* the Alexa 488 channel (λ_ex_ 488 nm, λ_em_ 500–540 nm).

For the endophilin binding experiments, GUVs conjugated with TIL were mixed with Alexa 594 labeled endophilin before imaging. A three-channel orthogonal imaging sequence was used to image in the Alexa 488 channel, Alexa 594 channel (λ_ex_ 561 nm, λ_em_ 580–620 nm), and DiD channel (λ_ex_ 640 nm, λ_em_ 650–750 nm) using three different detectors. The excitation lasers were alternately switched such that cross talk between different channels could be minimized. Images were analyzed with ImageJ and MATLAB programs. The fluorescence intensity in a given channel was quantified by fitting the GUV contour with a Gaussian ring (62).

### Determination of TIL density on the membrane

The TIL density was determined from the measured fluorescence intensity on the GUV surface from confocal images following an earlier established method (29, 30). To calibrate the fluorescence intensity *via* the mol% of a membrane bound fluorophore, GUVs composed of *x* mol% BODIPY-FL-DHPE and (100-*x*) mol% of DOPC, where x = 0.1 to 0.75, were imaged (at least ten GUVs for each mol%) under the same instrumental settings. A linear fit of the fluorescence intensity *versus* mol% plot (Fig. S9) gave rise to the relation,

Intensity=(201334±14039)x

Using the lipid headgroup area as 0.7 nm^2^ (29), from the above relation, the density of the BODIPY lipids = $\frac{\mathrm{Intensity}}{14\pm 1}$ molecules per μm^2^.

Considering Alexa-488 a two times brighter fluorophore than BODIPY (62, 63), the density of Alexa-488 is $\frac{\mathrm{Intensity}}{28\pm 2}$ molecules per μm^2^. From the Alexa-488 density, TIL density could be calculated as

$$\text{TIL density }=\;\frac{\mathrm{Intensity}}{\left(28\pm 2\right)e}\text{ molecules per }{\text{μm}}^{2}$$

where *e* is the labeling efficiency.

TIL-conjugated GUVs imaged under the same instrument settings resulted in a fluorescence intensity of 19,117 ± 7997 (mean ± sd from 15 independent GUVs). At the 50% labeling efficiency (*e* = 0.5), the TIL density resulted in 1400 ± 600 after rounding.

### Estimation of membrane area coverage by TIL

Membrane area coverage by TIL was determined from the density of TIL and the area occupied by single TIL molecule on the membrane. The area occupied by each TIL molecule was estimated from the theoretically predicted hydrodynamic radius (*R*_h_) of TIL using the following formula reported by Marsh and Forman-Kay as follows (48),

$$R_{h}=\left(AP_{pro}+B\right)\left(C\left|Q\right|+D\right)\;S_{His}\;R_{0}\;N^{\nu }$$

where *A* = 1.24, *B* = 0.904, *C* = 0.00759, *D* = 0.963, *S*_His_ = 1 (since there is no His-tag), *R*_0_ = 2.49, and *ν* = 0.509.

For TIL, using the values of the fraction of prolines *P*_pro_ = 16/80 = 0.2; number of residues, *N* = 80; and net charge |*Q*| = 13, this gave rise to *R*_h_ = 28.3 Å.

Using this value of *R*_h,_ the area occupied by single TIL molecule on the membrane (= π*R*_h_^2^) was obtained as 2.52 × 10^−5^ μm^2^

Considering, an average density of 1400 TIL molecules per μm^2^ membrane area, the area covered by TIL is ∼0.035 μm^2^ or ∼3.5% of the membrane area.

### Fluorescence recovery after photobleaching (FRAP) experiments

FRAP experiments were performed with a FluoView 3000 scanning system configured on an Olympus IX83 confocal microscope. TIL-Alexa 488-conjugated GUVs having lipid composition MCC-PE/DOPC/DiD (5:94.5:0.5) were first imaged by excitation with a 488 nm laser at 1% laser attenuation power. Photobleaching was performed at a selected box-shaped area on the GUV surface by applying 488 nm laser at 100% laser attenuation power for 1 s. Images of the recovery stage were collected immediately after the bleaching at a capture speed of 2.6 s per frame.

Images were analyzed with ImageJ software. For each time frame, mean intensity of a region of interest (ROI) chosen within the bleached region on the GUV surface was estimated and normalized against the mean intensity obtained from a similar ROI in the unbleached region. The normalized intensities were plotted with respect to time to obtain a FRAP recovery profile (Fig. S7*B*).

### Membrane tether pulling and analysis of curvature sorting

Membrane tethers were pulled from micropipette-aspirated GUVs composed of MCC-PE/DOPC/DiD/DSPE-PEG-Biotin (5:94.3:0.5:0.2 M ratio) (64, 65). Glass capillaries (World Precision Instruments (WPI, FL)) were pulled using a pipette puller (Sutter Instruments, CA) and the tips were remodeled with a microforge (WPI). Inner diameters of the capillaries were ∼7 μm for GUV aspiration and ∼5 μm for capturing beads. The capillary tips were passivated with β-casein solution (5 mg/ml). Capillaries were filled with HEPES buffer using a MicroFil needle (WPI) and fitted to the two arms of a motorized micromanipulator (Luigs & Neumann, Ratingen, Germany) oriented at an angle of 90º. One of these pipettes was used for aspiration of GUVs while a second one held a streptavidin-coated bead to pull tethers from the aspirated GUVs. Aspiration pressure was maintained by adjusting the height of a water reservoir connected to the pipette used for GUV aspiration, and the pressure was monitored by a pressure transducer (Validyne Engineering, Los Angeles, CA). Membrane tension (*T*) was determined from the aspiration pressure (Δ*P*), radius of the GUV (*R*_v_), and the radius of the projection area (*R*_p_) by using the formula (66),

$$T=\Delta P\times \frac{R_{p}}{2\left(1-\frac{R_{p}}{R_{v}}\right)}$$

TIL-conjugated GUVs were mixed with protein (Alexa 594-labeled or unlabeled endophilin) and pipette-aspirated. The aspiration pressure was maintained such that the resulting membrane tension was within the range of 0.12 ± 0.2 mN/m. The aspirated GUV was moved upward (by ∼ 0.5 mm) from the coverslip surface and incubated until the length of the aspirated vesicle projection stabilized. The tether was pulled by briefly (∼1 s or less) touching the streptavidin bead to the GUV surface and moving it to a distance of 12 to 15 μm from the surface carefully (at a speed of 2–3 μm/s approximately), in order to ensure that the tether remained intact.

The orientation and length of the pulled tether were adjusted by fine-tuning the x, y, and z positions of the bead with the micromanipulator system such that the tether was oriented along the center axis of the aspiration pipette and showed uniform fluorescence intensity along the tether length. Since DiD emission from the membrane shows a strong dependence on the laser polarization, all tethers were pulled at a fixed orientation with respect to laser polarization. Thus, the variation of DiD intensity on the tethers pulled from different GUVs is expected to be negligible.

Images were collected in Alexa 488, Alexa 594, and DiD channels. To determine the sorting coefficient, intensities on the flat GUV membrane were determined *via* a MATLAB script as described in previous sections. The aspirated region of the GUV was excluded from the fluorescence intensity analysis. The fluorescence intensity on the tether was quantified with ImageJ software by selecting a narrow, rectangular ROI covering the pixels along the length of the tether and measuring the mean pixel intensity within that ROI. Sorting coefficients were calculated using the formula (64):

$$\text{Sorting coefficient }=\;\frac{{\left(I_{tether}/I_{GUV}\right)}_{protein}}{{\left(I_{tether}/I_{GUV}\right)}_{lipid}}$$

FRAP experiments on the curvature-sorted endophilin-Alexa 594 were performed as described before. A small portion of the tether was bleached by applying 561 nm laser at 100% laser attenuation power. Images of the recovery stage were collected immediately after the bleaching at a capture speed of 4.7 s per frame.

### LUV preparation and TEM imaging

LUVs of desired lipid compositions were prepared following a method described elsewhere (19). Briefly, lipid films were formed by evaporating chloroform solutions of the desired lipid composition. HEPES buffer was added to the lipid films such that the resulting total lipid concentration was ∼1 mM. The aqueous suspension of lipids was vortexed and extruded through 400 nm pore-sized polycarbonate membranes (Whatmann/GE Healthcare) in order to obtain LUVs of uniform sizes. Size distribution of the LUVs was confirmed by dynamic light scattering (Malvern, United Kingdom) (Fig. S4). For the coupling of TIL, LUVs composed of MCC-PE/DOPC (5:95) were incubated (0.1 mM final lipid conc.) with TIL (250 nM) for 8 h at room temperature in HEPES buffer. The reaction was quenched by adding β-mercaptoethanol (5 mM).

For tubulation assays, LUVs (0.1 mM total lipid concentration) with or without conjugated TIL were incubated with endophilin (5 μM) in HEPES buffer for 30 min at room temperature. A droplet (20 μl) of the mixture was added onto a piece of parafilm, and a carbon-coated copper grid (Electron Microscopy Sciences, Hatfield, PA) was gently placed on the droplet such that the coated surface faced the liquid in order to allow the vesicles to stick to the grid. The grid was removed after 2 min, and excess solutions were soaked with a filter paper (Whatmann). The grid was washed (thrice for TIL-conjugated LUVs and once for LUVs having no TIL) by dipping into droplets of HEPES buffer, followed by removal of the excess buffer with a filter paper. For negative staining, the grid was placed on a droplet (20 μl) of 2% uranyl acetate solution for 2 min. Extra stains were washed thrice with buffer, and grids were kept on a filter paper for 10 min at room temperature for further drying. Images were recorded on a JEM 1011 transmission EM (JEOL, USA), operated at 100 kV, coupled with an ORIUS 832.10 W CCD camera (Gatan). Image analysis and postprocessing were performed with ImageJ software.

### Zeta potential measurements

Zeta potential measurements were performed on a Malvern Zetasizer Nano ZS device (Malvern, United Kingdom) using the folded capillary zeta cells (Malvern Panalytical Ltd, United Kingdom). LUVs of desired lipid composition were prepared by extrusion through 100 nm pore sized polycarbonate membranes (Whatmann/GE Healthcare). Before loading the sample, the cells were flushed with MilliQ water, followed by HEPES buffer. Measurements were taken using 0.1 mM lipids in 20 mM HEPES buffer containing 150 mM NaCl and 1 mM TECP; pH 7.4 at 25 °C. For TIL conjugation, LUVs (0.02 mg/ml) were reacted with TIL (100 nM) for 8 h at room temperature. The zeta potential of the TIL-conjugated LUVs was measured without further dilutions. The zeta potentials were estimated from the electrophoretic mobility using the Smoluchowski equation (67).

## Data availability

All data are contained within the article and the supporting information.

## Conflict of interest

The authors declare that they have no conflicts of interest with the contents of this article.
