# Supplementary material for: Endophilin recruitment drives membrane curvature generation through coincidence detection of GPCR loop interactions and negative lipid charge
Source: J Biol Chem. 2020 Dec 6;296:100140. doi: 10.1074/jbc.RA120.016118 (PMC7948419; doi:10.1074/jbc.RA120.016118)
Supplement: Supplementary Figures and Tables [file mmc1.pdf]

Supporting Information for

Endophilin recruitment drives membrane curvature generation through coincidence detection of GPCR loop interactions and negative lipid charge

Samsuzzoha Mondal, Karthik B. Narayan, Imania Powers, Samuel Botterbusch, and Tobias Baumgart*

* To whom correspondence may be addressed.
Email: baumgart@sas.upenn.edu

Supplementary Table:

Table S1: Zeta potentials (ζ) of LUVs composed of DOPC and MCC-PE

Lipid composition	ζ (mV)
DOPC	-3 ± 1
DOPC/DOPS (95:5)	-17 ± 2
DOPC/MCC-PE (95:5)	-18 ± 2
TIL + DOPC/MCC-PE (95:5)	-8 ± 1

Supplementary Figures:

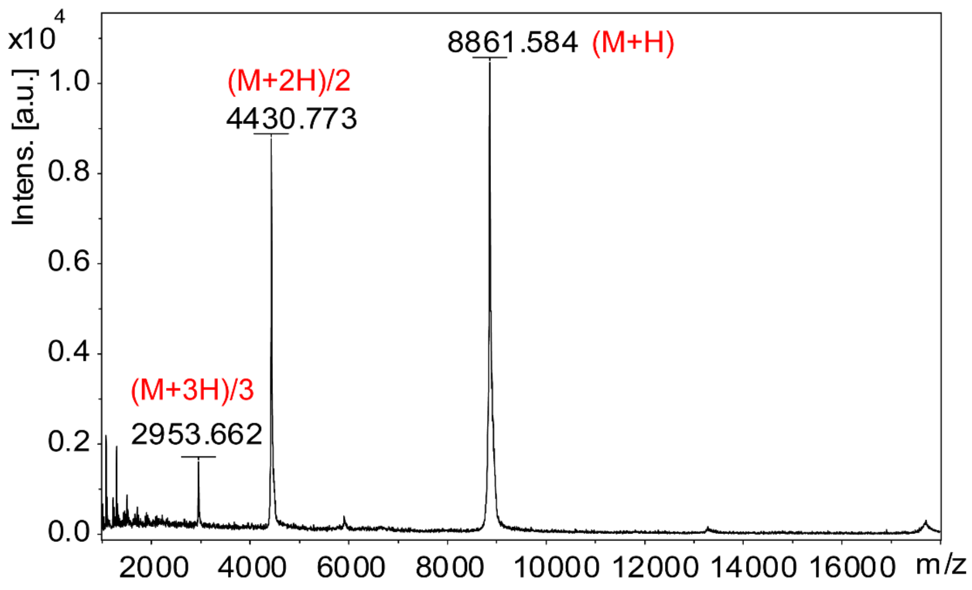

Figure S1. Characterization of purified TIL with MALDI mass spectrometry. Expected m/z: 8862 (M+H).

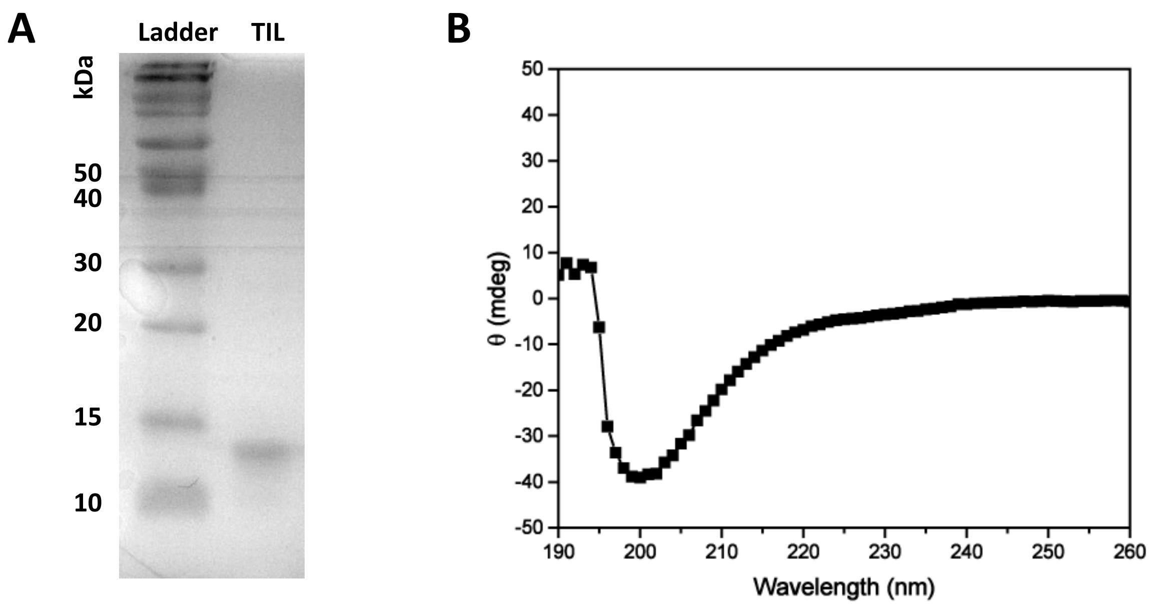

Figure S2. A. Characterization of TIL with SDS-PAGE. B. Circular dichroism (CD) spectra of purified TIL.

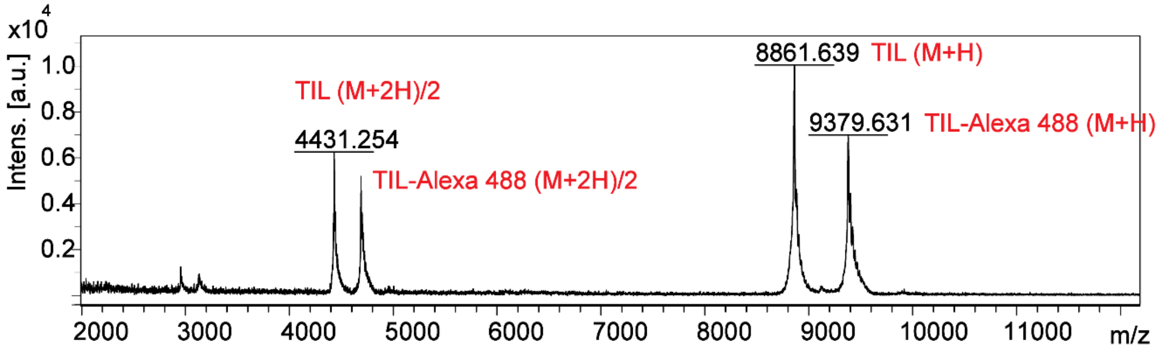

Figure S3. MALDI MS spectrum of TIL after labeling with Alexa 488-SDP ester. Expected m/z after labeling: 9379 (M+H).

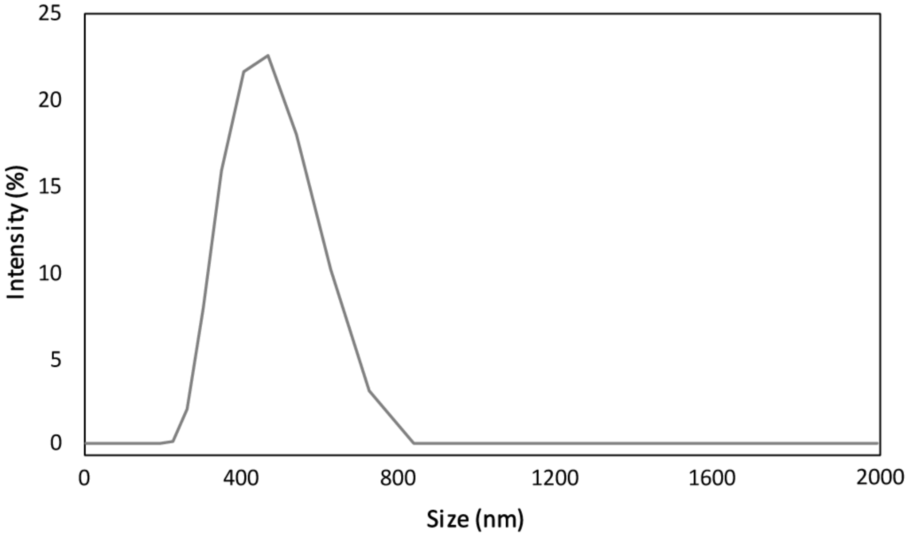

Figure S4. Size distribution of LUVs composed of 5% MCC-PE, 95% DOPC as obtained from dynamic light scattering.

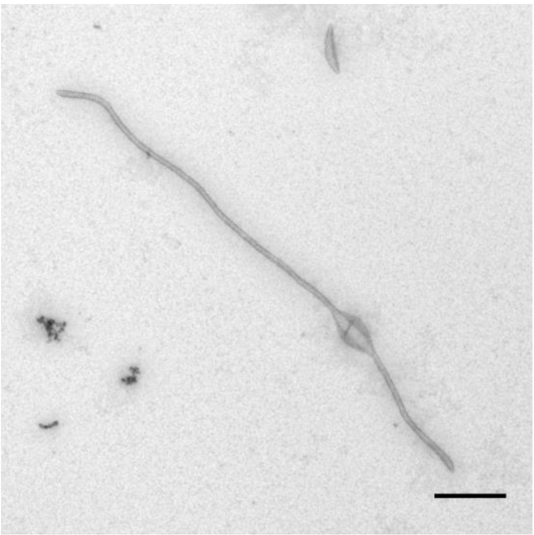

Figure S5. TEM image of a tubule formed by PS:PE:PC (45:30:25) LUV in the presence of endophilin (5 µM). Scale bar: 500 nm.

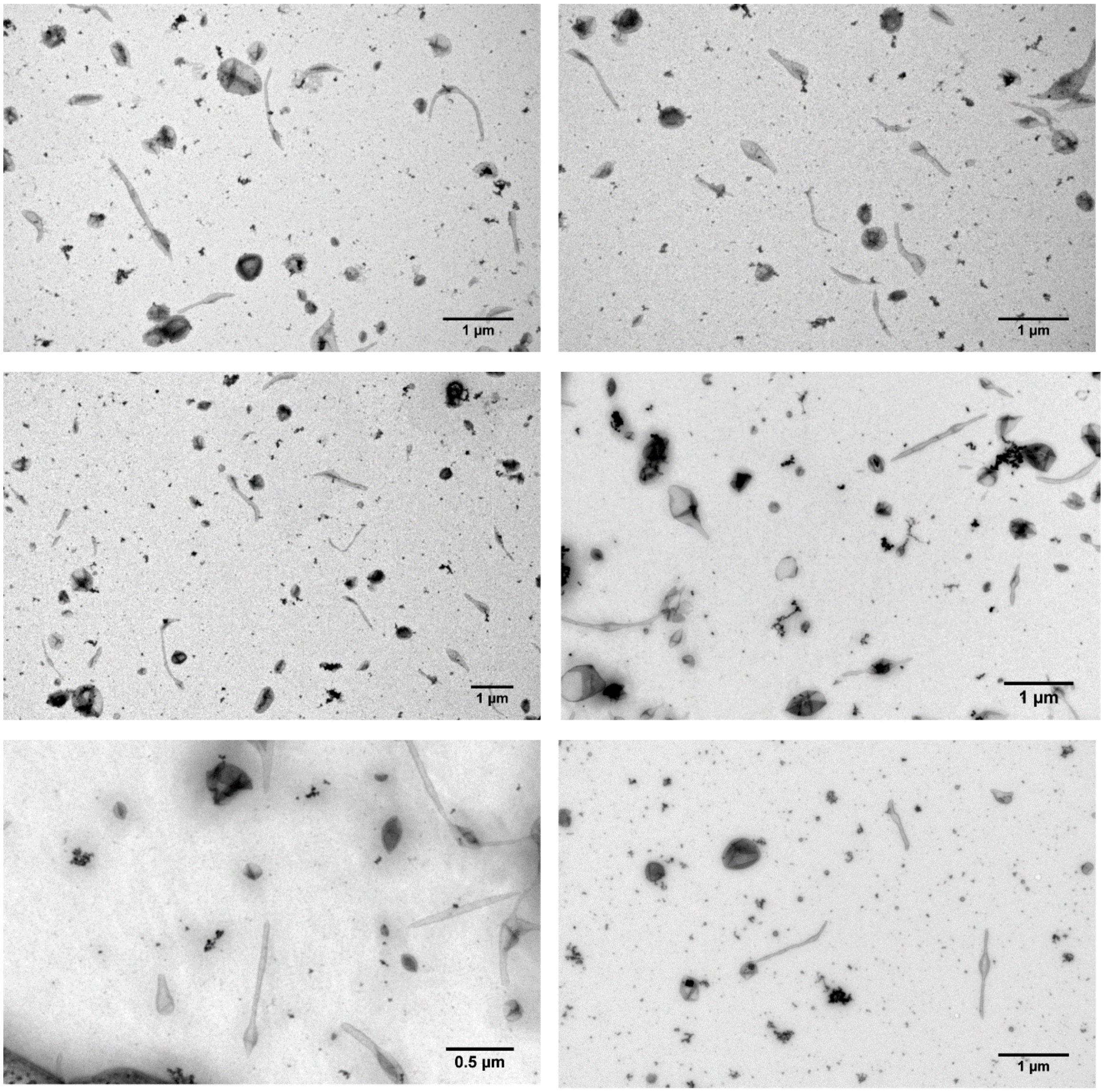

Figure S6. Extended dataset for TEM images of TIL-conjugated PC:MCC-PE (95:5) LUVs in the presence of endophilin (5 µM).

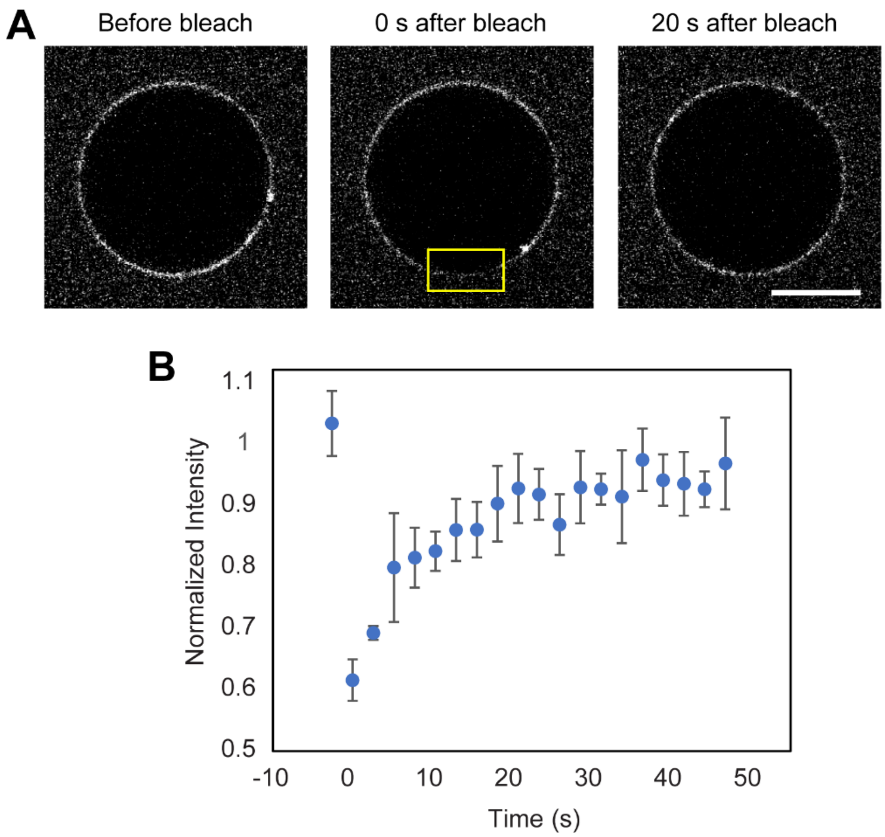

Figure S7. Fluorescence recovery after photobleaching (FRAP) studies on TIL-conjugated GUVs. A. Confocal images of TIL-conjugated GUVs (MCC-PE/DOPC/DiD) before, immediately after (0 s time delay) photobleaching, and 20 s after photobleaching. The yellow box indicates the membrane area irradiated for bleaching. Scale bar: 10 µm. B. Recovery profile obtained by plotting the normalized fluorescence intensities from the bleached membrane area with respect to time. Each data point is represented as mean ± standard deviation from five independent FRAP experiments.

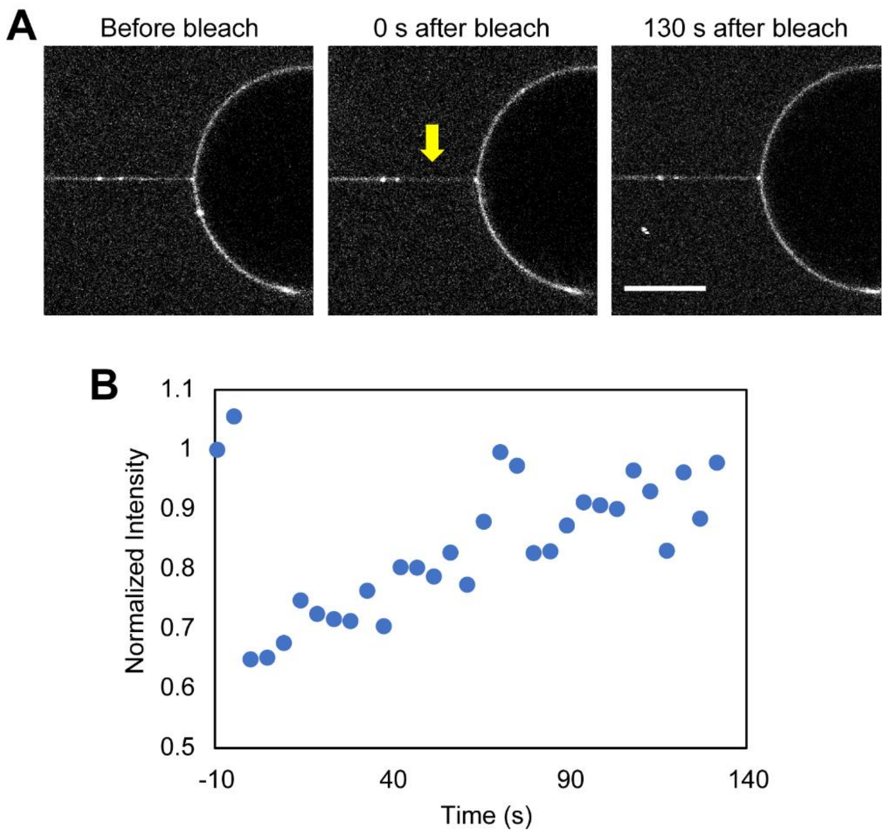

Figure S8. FRAP study to demonstrate the mobility of endophilin on a membrane tether. A. Confocal images of membrane-bound endophilin (Alexa 594 labeled) on a TIL-conjugated GUV with pulled tether. The images were recorded before, immediately after (0 s time delay) photobleaching, and 130 s after photobleaching. The arrow indicates the membrane tether area irradiated for bleaching. Scale bar: 10 µm. B. Recovery profile obtained by plotting the fluorescence intensities from the bleached tether area normalized against the intensity from an unbleached tether area with respect to time.

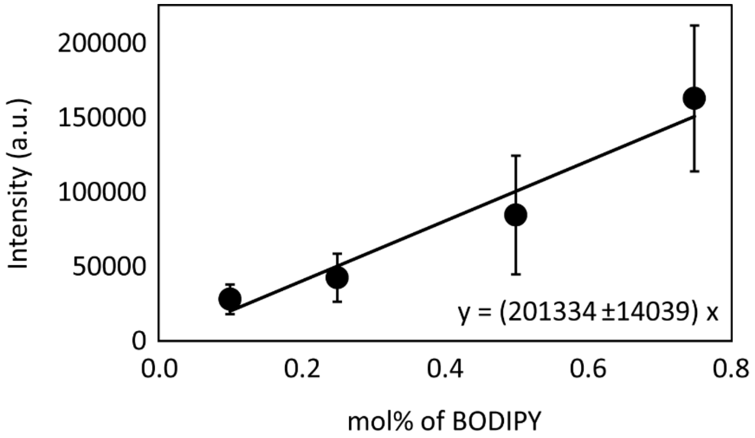

Figure S9. Fluorescence intensity calibration to estimate the protein-density on GUV membranes. GUVs composed of 0.1 to 0.75 mol% of BODIPY-FL-DHPE and DOPC were used and their fluorescence intensities were determined via confocal imaging. Each data point represents mean intensity ± standard deviation from at least 10 GUVs for the corresponding mol% of the BODIPY-lipid. The calibration equation was derived from a linear fit of the data points. The uncertainty value in the equation represents error of fitting (standard error).
